# Effect of Grip Type, Wrist Motion, and Resistance Level on Pressures within the Carpal Tunnel of Normal Wrists

**DOI:** 10.1002/jor.22571

**Published:** 2014-01-04

**Authors:** Raymond W McGorry, Nils Fallentin, Johan H Andersen, Peter J Keir, Torben B Hansen, Glenn Pransky, Jia-Hua Lin

**Affiliations:** 1 Liberty Mutual Research Institute for Safety71 Frankland Road, Hopkinton, Massachusetts, 01748; 2 Danish Ramazzini Centre, Department of Occupational Medicine, Regional Hospital HerningHerning, Denmark; 3 Department of Kinesiology, McMaster UniversityHamilton, Ontario, Canada; 4 Orthopaedic Research Unit, Regional Hospital HolstebroHolstebro, Denmark

**Keywords:** carpal tunnel syndrome, in vivo, pinch, power grip

## Abstract

Elevated carpal tunnel pressure (CTP) has been associated with carpal tunnel syndrome. This study systematically evaluated the effect of wrist motion resistance and grip type on CTP during wrist motion typical of occupational tasks. CTP during four wrist motion patterns, with and without resistance, and with and without gripping, was measured in vivo in 14 healthy individuals. CTP measured during compound motions fell between that measured in the cardinal planes of wrist flexion/extension and radial/ulnar deviation. Generally, with no active gripping there was little pressure change due to wrist angular displacement or resistance level. However, concurrent active pinch or power grip increased CTP particularly in motions including extension. CTP typically did not increase during wrist flexion, and in fact often decreased. Extension motions against resistance when employing a pinch or power grip increase CTP more than motions with flexion. Results could help inform design or modification of wrist motion intensive occupational tasks. © 2014 The Authors. © 2013 Orthopaedic Research Society. Published by Wiley Periodicals, Inc.

One mechanism for carpal tunnel syndrome (CTS) is believed to be elevated carpal tunnel pressure (CTP), the hydrostatic pressure that develops within the confined spaces of the carpal tunnel.^1^,^2^ Though the carpal tunnel is enclosed by bone and ligament circumferentially, it is an “open” anatomical compartment as it communicates with surrounding tissues proximally and distally. However, constrictions at the ends of the tunnel could occur due to incursion of muscle bellies into the tunnel ends (by the flexor digitorum superficialis and profundus proximally during wrist extension, or by the lumbricals distally during flexion of the metacarpal phalangeal joint).^3^,^4^ Swelling of the tenosynovium can occur due to prolonged exposure to repetitive, forceful activities, and local tissue swelling can disrupt blood supply, impairing nerve function. Tissue swelling and/or incursion of muscle bellies could restrict fluid flow and fluid pressures could rise due to the Bernoulli effect.

In animal studies, brief exposure to hydrostatic pressures of 30 mm of mercury (mmHg) slowed nerve conduction and produced paresthesias.^5^ Sustained hydrostatic pressures of 30 mmHg or greater may affect nerve physiology, and cause nerve damage and axon degeneration in animal models.^6^,^7^ In a study of experimentally induced median nerve compression (30–90 min duration) in healthy participants, 30 mmHg tunnel pressures caused hand paresthesias, and 60 and 90 mmHg pressures caused complete sensory and motor conduction block.^5^

There has been considerable interest in measuring pressures within the carpal tunnel to evaluate risks associated with hand/wrist work and activity. CTP has been measured using pressure transducers connected to catheters inserted into the carpal tunnel in studies spanning several decades.^8^,^9^ However, methodologies and instrumentation have varied greatly making comparisons among studies difficult. Despite such variability, within-study results have consistently demonstrated elevated CTP in wrists with CTS as compared to normal wrists. In wrists with CTS, tunnel pressures have been reported to exceed 30 mmHg when at rest in a neutral posture.^8^–^11^ In patients prior to undergoing surgery, CTP was typically elevated and returned to near normal levels following release of the transverse carpal ligament.^10^,^11^

Researchers have been successful in equating changes in CTP with wrist posture changes, forearm pro-/supination,^12^,^13^ wrist position in the flexion/extension and radial/ulnar planes, and finger and forearm posture.^8^,^14^,^15^ Using data collected coincident with CTP in healthy wrists, Keir et al.^16^ made the case for adopting work guidelines for wrist postures based on a 25–30 mmHg tunnel pressure threshold.

Knowledge of CTP response to force application at the wrist is less developed. Investigators have reported that in various activities force production with the fingers and hand can elevate tunnel pressures in both healthy wrists and those with CTS.^17^ Seradge et al.^18^ reported CTP elevation in healthy and CTS wrists when actively making a fist. A recent investigation with key and pulp pinch grips demonstrated increases in CTP with increasing grip force magnitude.^10^ Other reports have included work-related tasks; typing simulations, computer mouse use, pinching and finger pressing tasks.^19^,^20^

The above studies provide some insight as to how some activities affect CTP, under very specific conditions. How CTP changes with resistance over a range of working wrist postures, and further, how the gripping affects tunnel pressures during exposure to wrist motion patterns typical of industrial tasks is unclear.^16^ The present study was exploratory in nature, investigating the effect of the independent variables of wrist motion pattern, resistance to wrist motion, and type of grip application on the primary dependent in vivo measure of CTP, in healthy individuals with wrists moving through ranges of motion consistent with occupational exposures. A better understanding of the effect of these factors on CTP, in the context of wrist motion patterns typical of industrial tasks, can provide the basis for improving guidelines for a broader range of upper extremity work job and hand tool design.

## METHODS

### Participants

Fourteen right-handed participants (seven male), ages 20–35 with no recent history of upper extremity musculoskeletal disease were recruited. Participants gave written informed consent to participate in the study approved by the human subject protection committee of Region Midtjylland, Denmark. Participants' mean (SD) height, weight, and age were 175.5 (8.7) cm, 71.5 (9.4) kg, and 24.0 (3.0) years of age, respectively.

### Experimental Design

**Figure 1:**
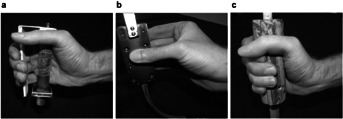
(a) The No-grip handle showing finger guard for finger positioning, (b) Pinch grip handle with a pinch pulp grip, and (c) The Power grip handle.

**Figure 2:**
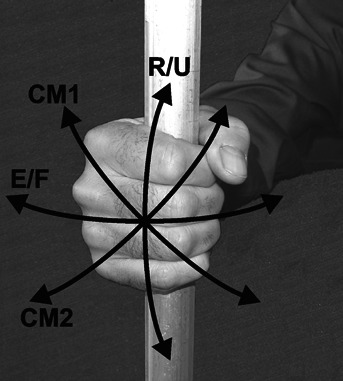
A photograph illustrating the four wrist motion condition: CM1—extension + radial deviation, and flexion + ulnar deviation, similar to a dart throwing motion; E/F—extension and flexion, CM2—extension + ulnar deviation, and flexion + radial deviation; R/U—radial and ulnar deviation.

A within-subject factorial design with three grip applications, no active gripping (No-grip), pinch grip (Pinch) and power grip (Power), were performed and analyzed independently. Figure 1 shows the three grip application conditions and the respective handles. Wrist motion levels and levels of resistance to wrist motion for each grip application were determined by pilot testing. Power grip (4 × 3) had four levels of wrist motion, defined as angular displacement in the wrist extension/flexion plane (E/F), in the radial/ulnar deviation plane (R/U), and two compound patterns. The first compound motion (CM1), similar to a dart throwing motion, involved motion about an axis of extension + radial deviation to flexion + ulnar deviation. The second compound motion (CM2) was about the axis of wrist extension + ulnar deviation to wrist flexion + radial deviation. Figure 2 illustrates the four motion patterns. Resistance to wrist motion for each of the grip conditions was provided by controlling the current to a brake in the experimental apparatus described below. Resistance levels were selected that would provide sufficient resistance to create separation between conditions, but at levels that could be accomplished without undue difficulty by the range of potential participants. Based on pilot testing three Power resistance levels were selected, 0, 1, and 2 N m. Pinch grip and No-grip conditions were only performed at two levels, 0 and 1 N m, as difficulty in performing the motions at the 2 N m was observed in pilot tests with smaller individuals. The Pinch grip application 3 × 2 had three motion levels, E/F, CM1, and CM2. The R/U motion was omitted from the Pinch grip condition because in pilot testing it was found that the motion was very awkward, and the range of motion so limited so as to be impractical to evaluate. No-grip (4 × 2), which involved no active gripping, had four wrist movement levels, E/F, R/U, CM1 and CM2, and two resistance levels.

### Instrumentation

#### Carpal Tunnel Pressure Measurement

The CTP measurement system included a 20 gauge (0.45 mm internal diameter) closed end, reinforced nylon, disposable multipore epidural catheter (Perifix™, B. Braun Melsungen AG, Melsungen, Germany). The 1-m long saline-filled catheter was connected via a three-way stopcock to a disposable fluid pressure transducer (Deltran™ DPT-100, Utah Medical Products, Midvale, Utah) with a measurement range from −30 to 300 mmHg, sensitivity of 5 µV/V/mmHg, and accuracy of ±1%. A normal saline IV bag hung approximately one meter above the wrist allowed for introduction of saline drops to keep the catheter tip clear. Similar methodologies have been previously reported.^8^,^21^ System calibration was verified in bench testing by water column to 220 mmHg, prior to and following data collection.

#### The Experimental Apparatus

**Figure 3:**
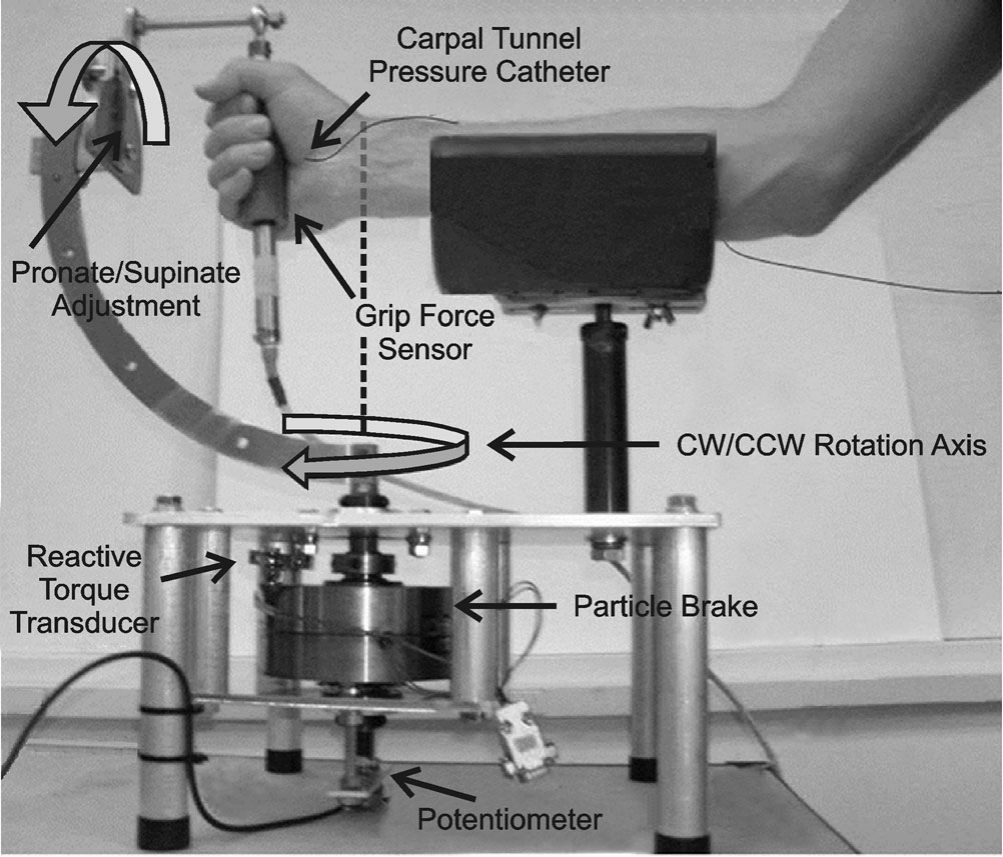
The experimental apparatus detailing the pivot and lock mechanism for adjusting the handle orientation, the magnetic particle brake that provides resistance to clockwise and counterclockwise motion about the axis aligned with the wrist, and the potentiometer that measures wrist angular displacement. The dashed vertical line illustrates alignment of the particle brake shaft with the wrist center of rotation.

Figure 3 illustrates the components of the experimental apparatus. The system was constructed allowing for real-time control of wrist motion resistance and display of wrist angular displacement, grip force, and CTP. A locking pivot allowed for setting the four wrist motion levels. A magnetic particle brake provided 0, 1, and 2 N m resistances around a shaft aligned with the wrist axis, as indicated by the dashed vertical line in Figure 3. Also as seen in Figure 3, the pronate/supinate adjustment allows the forearm to be positioned in: neutral, 90° pronation, 45° supination, and 45° pronation. In each position, with the wrist aligned with the particle brake axis, active wrist movement corresponded to the motion levels E/F, R/U, CM1, and CM2, respectively. The potentiometer on the particle brake shaft measured wrist angular displacement for the respective motion level. CTP, grip force, wrist motion resistance, and angular displacement signals were sampled at 50 Hz and stored on a personal computer. The handles were instrumented with strain gauges (6 gauges in the Power grip handle and 4 gauges in the Pinch grip handle) configured to resolve applied forces to thus allowing the experimenters to monitor grip application. A 40 mm diameter handle was fabricated for Power grip, a 1.5 cm thick rectangular (4 cm × 8 cm) handle, for Pinch grip. The No-grip handle had a finger guard that maintained consistent finger positioning while preventing assistance by the fingers, thus allowing movement against resistance without the confounding effects of gripping.^16^,^19^

### Experimental Procedure

#### Catheter Insertion

Under local anesthesia (lidocaine, 10 mg/ml), a small transverse incision was made at the distal volar crease by a hand surgeon. A plastic cannula inserted through the flexor retinaculum was used to guide the catheter into the carpal tunnel. The catheter was advanced to the hook of the hamate and the cannula retracted. The surgical site was sealed with a waterproof film. The catheter was flushed with four drops of normal saline. Response of the pressure measurement system was verified. The catheter was introduced between the median nerve and the flexor retinaculum with the tip introduced to the level of the hook of the hamate. The location was selected because it is a site common to multiple reports in the literature, provides a unique bony landmark verifiable on imaging, and is a site often reported as having higher tunnel pressures.^8^,^10^,^18^,^22^ The location of the catheter tip within the carpal tunnel was verified with ultrasonic imaging.

#### Protocol

The participant sat with the right elbow flexed 90° and the shoulder slightly abducted from the side. The right forearm was placed in the cradle of the apparatus and the long axis of the forearm was aligned with the center of the pronation/supination pivot, and the ulnar styloid was aligned with the center of the clockwise (CW)/counterclockwise (CCW) rotation axis (Fig. 3). Foam wedges placed beside of the forearm minimized forearm movement and maintained alignment.

The three grip applications were performed in independent blocks starting with Power, followed by Pinch and No-grip. At the start of each block, CTP was recorded with the wrist and forearm relaxed, in the forearm support in the neutral posture for the motion to subsequently be tested. Within block, wrist motion and resistance level order were randomly assigned. Participants were given several motion cycles to familiarize themselves with each motion and resistance level. The instructions were to move the handle smoothly and slowly through a comfortable range of motion. For No-grip, the two bars of the handle were adjusted to gently clamp the palmar and dorsal surfaces of the metacarpal phalangeal (MCP) joints. The hand could push the handle with the dorsal or palmar aspect of the MCP joints without active gripping, yet maintain consistent finger position by resting the fingers against the flexible plastic guard that offered no purchase for applying force. For the Power and Pinch conditions, participants were given additional instruction and training. Participants were instructed to grasp the handle firmly as one might do during a precision task, gripping as necessary to control the handle but without using excess force, and to do so as consistently as possible across trials. During practice and experimental trials gripping was verified in real time by experimenter observation of the instrumented handle output. The neutral posture was determined prior to the first trial of each grip application block by viewing the arm from above and aligning the center of the handle with the long axis of the radius. The posture was used as the reference for all trials within the block. Starting from neutral participants were instructed to move slowly and smoothly within their comfortable range, through two full cycles of wrist motion, thus moving through their range of motion twice in each the clockwise and counterclockwise directions. One to two drops of saline were introduced intermittently to keep the catheter tip clear. No more than 1 ml of saline was introduced while the catheter was indwelling (<2 h). At the end of the protocol the hand surgeon withdrew the catheter and dressed the insertion site. No adverse effects were reported due to the experimental procedure.

### Data Processing and Analysis

To ensure that data reflected smooth, slow, dynamic motion, the data from the beginning and ending half-cycles of the trial was discarded to avoid variability associated with initiating motion or overcoming the initial static friction, thus providing two wrist motions (one CW, one CCW) for each condition. For each participant angular displacement data for each trial was aligned at the neutral (0°) position. Within each grip block and for each wrist motion level, CTP at the neutral position for the 0 N m trial was treated as the reference. That value was subtracted from all CTP values at all resistance levels, in effect standardizing all pressures to the neutral-no load state. Standardized range of motion data for all participants was then averaged in five-degree bins, referred to as “segments,” and individual and group mean segment values were calculated. Paired *t* tests of the mean CTP for each five-degree segment were compared to the neutral-no load condition. Significance level was set at *P* < 0.05. Mean CTP across motion segments was calculated for all trials, for 14 participants for the No-grip and Power grip conditions, and 13 participants for the Pinch grip condition (one subject did not complete the block due to technical problems). For each grip application data were collapsed across resistance levels, and means were calculated for each motion pattern.

## RESULTS

The overall mean (SD) CTP at rest was 3.5 (2.3) mmHg, range 0–8.7 mmHg. For all participants, collapsing results from all trials of all grip applications, the overall mean pressure was 8.8 (5.6) mmHg. The smallest individual overall mean pressure was 4.6 mmHg, and the largest was 26.2 mmHg. For any one participant, the smallest range of pressures observed across all trials of any grip application was 0–6.0 mmHg occurring during Pinch grip. The largest range of pressures for one participant was 0–49.0 mmHg observed during Power grip.

Summary data for the four motion levels and three grip applications collapsed across participant and resistance levels are presented in Table1.

**Table 1:** Mean (SD) carpal tunnel pressures (mmHg) for the four motion levels and three grip conditions, collapsed across participant, and resistance level

Motion Level	Motion Pattern	No-Grip	Power Grip Means (SD) (mmHg)	Pinch Grip
CM1	Ext & Radial Dev/Flex & Ulnar Dev	10.4 (10.1)	10.6 (9.3)	11.1 (10.0)
E/F	Extension/Flexion	6.9 (6.5)	9.7 (9.9)	9.8 (7.8)
CM2	Ext & Ulnar Dev/Flex & Radial Dev	7.6 (7.2)	7.7 (7.6)	11.7 (9.3)
R/U	Radial/Ulnar Deviation	9.4 (10.8)	6.0 (7.8)	

**Figure 4:**
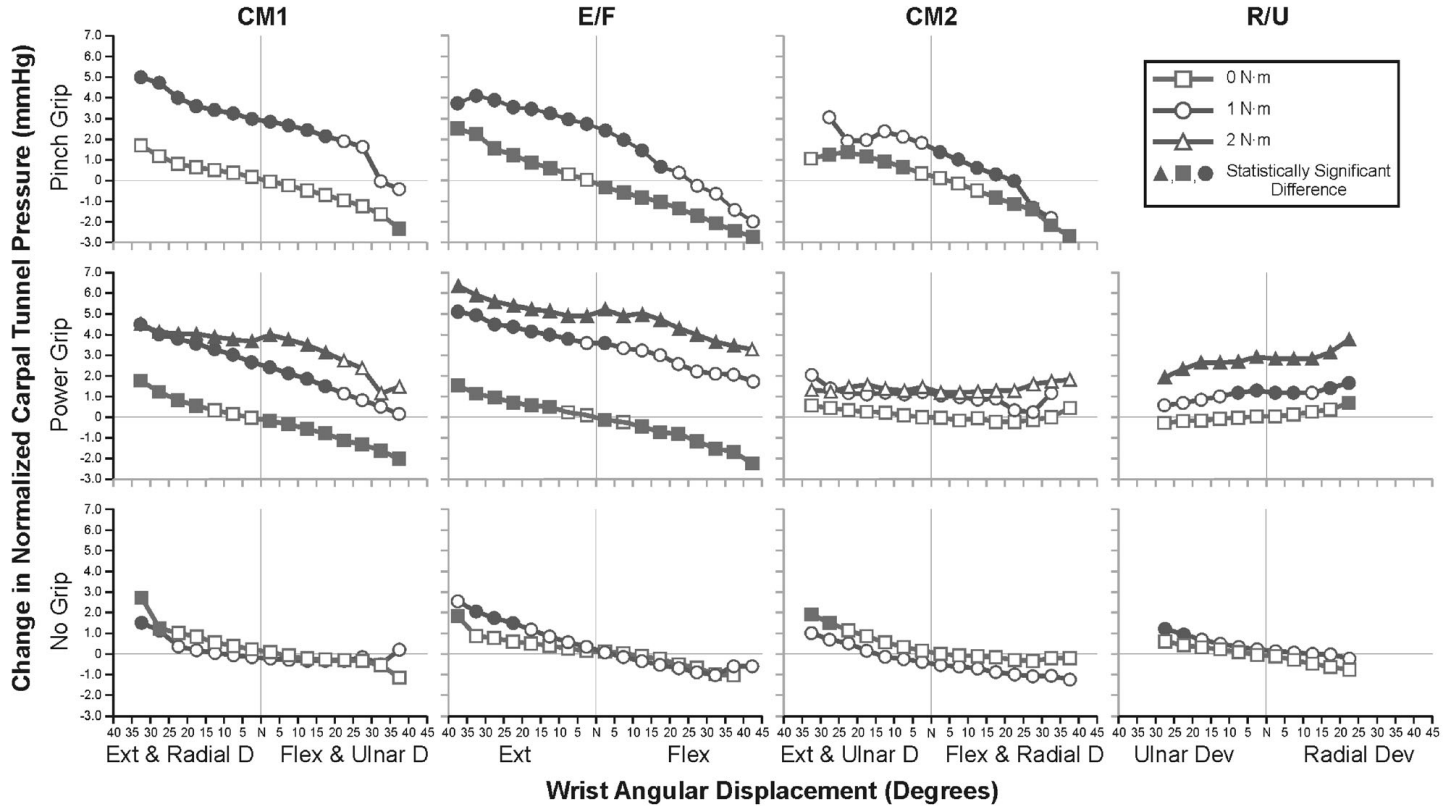
Carpal tunnel pressure (*y*-axis) versus wrist angular displacement (*x*-axis), each graph with a family of curves of resistance levels. By rows: Pinch Power and No-grip applications. By column, wrist motion conditions, left to right: (CM1)—extension/radial deviation & flexion/ulnar deviation, (E/F)—extension & flexion, (CM2)—extension/ulnar deviation & flexion/radial deviation, (R/U)—radial deviation & ulnar deviation. Sold-filled symbols indicate values significantly different from the neutral no-load condition.

Data for each motion segment was standardized, as described in Methods, to allow investigation of change in CTP across participants, and to allow for quantifying tunnel pressure change as a function of wrist angular displacement and resistance level from the neutral, no-load condition. The results of the analyses of standardized change in CTP by wrist angular displacement for each grip application are presented in Figure 4, with solid symbols indicating significantly different pressures from the neutral, no-load condition. Resistance levels are presented as a family of curves.

Of the three grip conditions, the No-grip condition produced the lowest overall tunnel pressures, and without gripping the response to resisted wrist motion was generally small. The Pinch grip produced the greatest overall CTP, being at least 18% greater than with Power grip or No-grip conditions. With either the pinch grip or power grip there was generally a greater increase in CTP in response to resisted wrist motion than observed when no active gripping was used.

## DISCUSSION

The resting pressure in neutral wrist posture is a measure reported in many CTP studies. In the present study, the overall mean (SD) CTP at rest was 3.5 (2.3) mmHg, range 0–8.7 mmHg. This result agrees, within 1 SD, of results reported in several studies.^9^,^15^,^22^,^23^ Two other studies have reported higher resting neutral pressures, ranging from 7.2 mmHg to a high of 24 mmHg.^18^,^24^ It is not clear whether this variability is due solely to normal variance within the healthy population, or if there is a contribution due to differences in methodology.

Several findings relevant to those studying repetitive upper extremity occupational tasks resulted from the investigation. Changes in tunnel pressures with resistance to wrist motion evaluated in a controlled fashion through a large range of wrist motions were quantified in a population of healthy wrists. To our knowledge, this study is also the first to report on CTP changes in response to motions other than the cardinal wrist motion planes of flexion/extension or radial/ulnar deviation. Overall the data suggests that when compound motions are involved, as would be typical of industrial activities, pressures within the carpal tunnel tend to fall between those observed in the cardinal planes of wrist flexion/extension and radial/ulnar deviation.

The results also showed that CTP changed with different grip applications, and pressures were generally greater with active pinch and power grips as compared to trials where there was no active gripping. Overall, the No-grip condition produced the lowest CTP. In Figure 4, it is notable that, in the absence of concurrent gripping, the response to 1 N m of wrist motion resistance was minimal. There were some significant increases in CTP at the extremes of the four motion levels but the magnitude of the differences was small and likely of little practical significance.

The response to wrist angular displacement was more pronounced when there was active gripping. With respect to Pinch, overall pressures were at least 18% greater than in the No-grip or Power condition. When examining the Pinch grip data two trends become clear. Most notably there was a consistent trend toward increased CTP with 1 N m of resistance compared to the no-load state. The other trend to note was the increase in CTP with wrist extension or the compound extension motions of CM1 and CM2, though these trends were not statistically significant under the no-resistance state for CM1 and the 1 N m resistances for CM2. CTP generally decreased in a majority of motion segments during wrist flexion under no load and for CM2 under both resistance levels.

There were three resistance levels for Power grip, and for three of the motion levels, excluding CM2, there was a clear differentiation in pressure response to resistance. The response was strongest in extension/flexion. With CM1 and extension/flexion motions the trending with angular displacement was similar to Pinch. With the compound motion of extension with ulnar deviation & flexion with radial deviation, CM2, there were no significant responses to angular displacement at any level, a response similar to No-grip. In the radial/ulnar deviation motion plane, in addition to the monotonic increase in pressure with resistance CTP increased at the extremes of radial deviation, particularly at 2 N m.

The study investigated CTP with respect to wrist angular displacement with and without resistance over a range of motion typical of occupational tasks. The trend of increasing CTP with wrist extension was typical of that reported in the literature.^15^,^16^,^24^ Perhaps the most striking difference between the present findings and previous reports were the trends observed during wrist flexion. CTP tended to decrease when wrist flexion was a component of the motion pattern and there was active gripping. Previous studies of both normal and CTS wrists have generally reported increasing CTP with wrist flexion, particularly at end ranges as great as 50°.^15^,^16^,^24^ Those studies did not involve gripping concurrent with resisted wrist motion, and end ranges were greater than that evaluated in the present study.

While differences in methodology and instrumentation may account for some of the differences in these observations, there may be an additional factor at play. CTP may vary by location within the tunnel in normal wrists^17^ and in CTS cases.^10^,^17^ Pressures are reported to be greater distally, at least as distal as the hook of the hamate. We speculate that differences in catheter insertion locations may affect behaviors in the CTP measures as reported in the literature. Insertion locations reportedly range from at the distal volar crease (the present study) to as far as 3 cm proximal to the distal volar crease.^11^ Inserting the catheter and anchoring it at the distal volar crease, the assumed center of rotation, should minimize longitudinal excursion within the tunnel and thus more accurately reflect CTP during wrist flexion and extension motion. A more proximal insertion would likely permit greater movement of the tip within the tunnel during wrist motion, potentially causing pressure changes secondary to tip location. Imaging studies of the catheter tip location in vivo using various insertion sites might be warranted to address this methodological concern.

Standardization of CTP was adopted based on the observation of large individual variation in responsiveness of tunnel pressures. Across the range of experimental conditions, the CTP for one participant ranged over only 6 mmHg, while another participant experienced an eightfold greater range of response. Large individual differences in CTP have similarly been reported in the literature.^16^

It is also difficult to compare absolute CTP values across studies due to differences in methodology and instrumentation. For example, CTP for healthy wrists moved passively to full wrist extension have reportedly ranged from 13 mmHg^23^ to nearly 158 mmHg.^11^ Actively making a fist reportedly produced a mean CTP of 234 mmHg in 21 healthy wrists.^18^ Such large variations are likely due to multiple factors. CTP studies have utilized various sensor technologies. Fluid filled catheters systems with external pressure transducers are the most common measurement technology reported, but even these systems have utilized different catheter designs including wick tip catheters,^9^ slit tip catheters,^22^ an angiocath,^11^ and multipore epidural catheters.^8^,^21^ Another study used catheters with strain gauges^10^ that respond to local contact pressure as well as fluid pressures. Experimental protocols also vary greatly, some reporting on pressures from actively maintained postures, passive positioning, and varying finger position; all of which are factors reported to modulate pressure. While standardization facilitates analysis by comparing CTP recorded during experimental conditions to a neutral-no load state within a study, adoption of this approach could simplify comparison of results between studies. Generalizability would likewise be enhanced by adoption of standards for insertion sites, and instrumentation for CTP measurement.

In conclusion, the study provided the first systematic analysis of the effects of grip application, resistance level and compound wrist motion on carpal tunnel pressures in normal wrists moving through normal ranges of movements. Increases in CTP with increased resistance to wrist motion were observed under some wrist motions, and some segments of angular displacement, but not with others. The compound wrist motions tested in this study in general tend to behave in intermediate fashion to the cardinal plane motions typical of most previous studies. Perhaps the most important and unexpected finding was that while the results reinforce prior findings that wrist extension against resistance while gripping may increase CTP, flexion motions generally did not, and under some conditions pressure levels decreased significantly. Another practical consideration that can be gleaned from the results is that design or modification of occupational tasks with a focus on reducing grip force, and the avoidance of wrist extension postures in tasks requiring gripping, could reduce tunnel pressures and thus the potential associated risk.

Like most investigations, the results raise questions deserving further consideration, and there are limitations to consider prior to generalizing the results of this study. Though grip force application was monitored in this study, it was not completely controlled. Secondly, participants had normal wrists, and how pressures in wrists with CTS might respond cannot be construed with certainty. It could prove informative to investigate how the CTP of individuals with demonstrable CTS or early symptoms of the disease respond to similar experimental conditions. Verification of the catheter tip location within the tunnel during wrist movement could not be assured without use of real-time imaging, though the technique of introducing the catheter at the distal wrist crease employed in this study could minimize displacement. Further investigation of how catheter insertion point affects CTP measures with respect to wrist motions including flexion seems warranted considering differences between the present study results and previous reports. Finally, the wrist motions employed in the protocol were slow and smooth, and gripping was relatively static. Further studies of the effect of duty cycle in repetitive gripping on the dynamics of pressure development within the carpal tunnel could provide data useful to evaluating repetitive occupational tasks, and could have implications for job design.

## References

[1] Keir PJ, Rempel DM (2005). Pathomechanics of peripheral nerve loading. Evidence in carpal tunnel syndrome. J Hand Ther.

[2] Rempel DM, Diao E (2004). Entrapment neuropathies: pathophysiology and pathogenesis. J Electromyogr Kinesiol.

[3] Keir PJ, Bach JM (2000). Flexor muscle incursion into the carpal tunnel: a mechanism for increased carpal tunnel pressure. Clin Biomech.

[4] Yii NW, Elliot D (1994). A study of the dynamic relationship of the lumbrical muscles and the carpal tunnel. J Hand Surg Br.

[5] Lundborg G, Gelberman RH, Minteer-Convery M (1982). Median nerve compression in the carpal tunnel–functional response to experimentally induced controlled pressure. J Hand Surg Am.

[6] Diao E, Shao F, Liebenberg E (2005). Carpal tunnel pressure alters median nerve function in a dose-dependent manner: a rabbit model for carpal tunnel syndrome. J Orthop Res.

[7] Lim JY, Cho SH, Han TR (2004). Dose-responsiveness of electrophysiologic change in a new model of acute carpal tunnel syndrome. Clin Orthop Relat Res.

[8] Coppieters MW, Schmid AB, Kubler PA (2012). Description, reliability and validity of a novel method to measure carpal tunnel pressure in patients with carpal tunnel syndrome. Man Ther.

[9] Gelberman RH, Hergenroeder PT, Hargens AR (1981). The carpal tunnel syndrome. A study of carpal canal pressures. J Bone Joint Surg Am.

[10] Goss BC, Agee JM (2010). Dynamics of intracarpal tunnel pressure in patients with carpal tunnel syndrome. J Hand Surg.

[11] Okutsu I, Ninomiya S, Hamanaka I (1989). Measurement of pressure in the carpal canal before and after endoscopic management of carpal tunnel syndrome. JBJS Am.

[12] Rempel DM, Bach JM, Gordon L (1998). Effects of forearm pronation/supination on carpal tunnel pressure. J Hand Surg Am.

[13] Werner R, Armstrong TJ, Bir C (1997). Intracarpal canal pressures: the role of finger, hand, wrist and forearm position. Clin Biomech.

[14] Weiss ND, Gordon L, Bloom T (1995). Position of the wrist associated with the lowest carpal-tunnel pressure: implications for splint design. J Bone and Joint Surg.

[15] Keir PJ, Bach JM, Rempel DM (1998). A. Effects of finger posture on carpal tunnel pressure during wrist motion. J Hand Surg Am.

[16] Keir PJ, Bach JM, Hudes M (2007). Guidelines for wrist posture based on carpal tunnel pressure thresholds. Human Factors.

[17] Luchetti R, Schoenhuber R, De Cicco G (1989). Carpal tunnel pressure. Acta Orthop Scand.

[18] Seradge H, Jia Y-C, Owens W (1995). In vivo measurement of carpal tunnel pressure in the functioning hand. J Hand Surg Am.

[19] Keir PJ, Bach JM, Rempel DM (1998). Fingertip loading and carpal tunnel pressure: differences between a pinching and pressing task. J Orthop Res.

[20] Rempel DM, Keir PJ, Bach JM (2008). Effect of wrist posture on carpal tunnel pressure while typing. J Orthop Res.

[21] Rempel DM, Manojlovic R, Levinsohn DG (1994). The effect of wearing a flexible wrist splint on carpal tunnel pressure during repetitive hand activity. J Hand Surg Am.

[22] Szabo RM, Chidgey LK (1989). Stress carpal tunnel pressures in patients with carpal tunnel syndrome and normal patients. J Hand Surg Am.

[23] Rojviroj S, Sirichativapee W, Kowsuwon W (1990). Pressures in the carpal tunnel. A comparison between patients with carpal tunnel syndrome and normal subjects. J Bone Joint Surg Br.

[24] Rempel D, Keir PJ, Smutz WP (1997). Effects of static fingertip loading on carpal tunnel pressure. J Orthop Res.

